# PRR34-AS1 promotes exosome secretion of VEGF and TGF-β via recruiting DDX3X to stabilize Rab27a mRNA in hepatocellular carcinoma

**DOI:** 10.1186/s12967-022-03628-9

**Published:** 2022-10-27

**Authors:** Zhilei Zhang, Ye Zhou, Yuming Jia, Chao Wang, Meng Zhang, Zhuo Xu

**Affiliations:** Department of Hepatobiliary Surgery, Fourth Hospital of Hebei Medical University, No. 12 Jiankang Road, Shijiazhuang, 050011 Hebei China

**Keywords:** Hepatocellular carcinoma, PRR34-AS1, Exosome, Rab27a

## Abstract

**Background:**

Exosomes are deemed to be an important tool of intercellular communicators in cancer cells. Our study investigated the role of PRR34 long non-coding RNA antisense RNA 1 (PRR34-AS1) in regulating exosome secretion in hepatocellular carcinoma (HCC) cells.

**Methods:**

Quantitative real-time polymerase chain reaction (RT-qPCR) analyzed the expression of PRR34-AS1. We assessed the function of PRR34-AS1 on the biological changes of THLE-3 cells and HCC cells. The downstream interaction between RNAS was assessed by mechanistic experiments.

**Results:**

PRR34-AS1 expression was upregulated in HCC cells in comparison to THLE-3 cells. PRR34-AS1 depletion repressed HCC cell proliferation, migration and invasion as well as EMT phenotype, while PRR34-AS1 up-regulation accelerated the malignant phenotypes of THLE-3 cells. PRR34-AS1 recruited DDX3X to stabilize the mRNA level of exosomal protein Rab27a. Moreover, PRR34-AS1 facilitated the malignant phenotypes of THLE-3 cells by elevating Rab27a expression to promote the exosome secretion of VEGF and TGF-β in HCC cells.

**Conclusions:**

The current study revealed a novel function of PRR34-AS1 in accelerating exosome secretion in HCC cells and offered an insight into lncRNA function in the regulation of tumor cell biology.

**Graphical Abstract:**

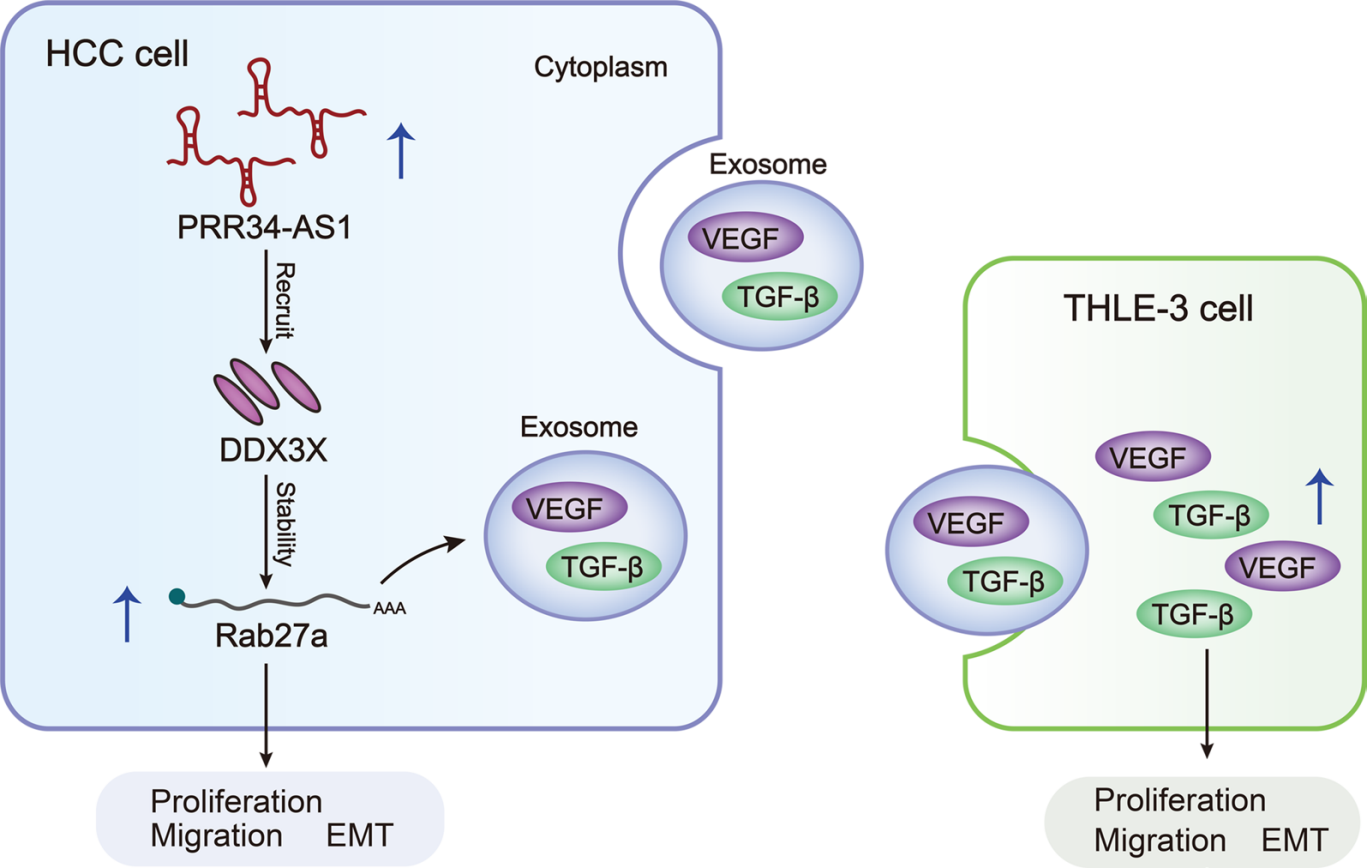

**Supplementary Information:**

The online version contains supplementary material available at 10.1186/s12967-022-03628-9.

## Background

Hepatocellular carcinoma (HCC) is one of the most common malignancies worldwide, with a high morbidity and mortality rate [1, 2]. Surgical resection is identified as the most effective therapy for HCC treatment, but high recurrence and distant metastasis after surgery result in poor prognosis of HCC [3, 4]. Exosomes play important roles in multiple aspects of HCC, which include angiogenesis, chemoresistance and metastasis [5]. Moreover, some exosome biomarkers are used for the early diagnosis and prognosis of HCC patients [6, 7]. Cancer cell-secreted exosomes were considered as important messengers in intercellular communication, thus many studies have unveiled the function of exosome in tumor microenvironment [8]. Nevertheless, few studies have addressed the underlying molecular mechanisms of tumor cell secretion of exosomes.

Exosomes are 30–150 nm nano-sized vesicles containing various types of nucleic acids, such as proteins, RNAs and lipids [9]. Exosome biogenesis is formed by the budding of multivesicular body (MVB) membranes inwards to shape intra-luminal vesicles, ultimately maturing and contained within MVBs [10]. Multiple effectors and molecular mechanisms are involved in regulating the intracellular trafficking steps such as MVB movement, docking, and integration of exosomes with the plasma membrane before release [11]. Rab GTPases are responsible for regulating MVB motility and plasma membrane docking [12]. The Rab family contains many subtypes, and some Rab GTPases are crucial mediators in modulating exosome secretion, such as Rab27A and Rab27B. Rab27A and Rab27B have been reported to be the primary components of vesicle trafficking in exosome secretion and play critical roles in tumor progression and metastasis [13]. Nevertheless, the mechanisms by which Rab GTPases control the secretion of exosome in HCC cells remain to be further investigated.

According to global gene expression data in mammalian species, the majority of the genome is transcribed into non-coding RNAs (ncRNAs) [14]. Long non-coding RNA (lncRNA) is a class of ncRNAs greater than 200 nucleotides in length, and has been emerged as pivotal regulators in cancer progression [15]. LncRNAs can regulate gene expression through transcriptional regulation, post-transcriptional regulation, chromatin modification as well as genomic imprinting [16]. Aberrantly expressed lncRNAs can participate in HCC progression as oncogenes or tumor suppressors [17]. MCM3AP-AS1 exerts an oncogenic role in HCC progression via interacting with miR-194-5p to elevate FOXA1 expression [18]. MAGI2-AS3 impedes cell proliferation and migration in HCC via the miR-374b-5p/SMG1 axis [19]. LncRNA PRR34 long non-coding RNA antisense RNA 1 (PRR34-AS1) regulates HCC cells malignant phenotypes through miR-296-5p/SOX12/E2F2 axis [20] or miR-498/TOMM20/ITGA6 axis [21].

In this study, to further reveal the effect of PRR34-AS1 in HHC, we probed into the role of PRR34-AS1 in HCC, and investigated the mechanism between HCC cells and THLE-3 cell by exosome secretion. Our study might offer a novel sight for HCC treatment.

## Methods

### Cell culture

HCC cells including HLF, Huh-7, SNU-449, HepG2 and LM3 were obtained from COBIOER (Nanjing, China). HLF, Huh-7 and LM3 cells were grown in DMEM. SNU-449 cells were grown in RPMI-1640 medium. HepG2 cells were grown in MEM. All mediums were obtained from Gibco (Grand Island, USA). Human liver epithelial cell (THLE-3) was obtained from ATCC (Manassas, VA, USA) and kept in BEGM (Lonza/Clonetics Corporation, Walkersville). Cells were left to grow at 37 °C under a humid environment with 5% CO_2_.

### Quantitative real-time PCR (RT-qPCR)

Total RNAs were extracted with the application of Trizol reagent (Invitrogen, USA). For the evaluation of gene expression, cDNA was synthesized with the application of PrimeScript RT master mix (Takara, Japan). Next, SYBR Green PCR Master Mix (Applied biosystems) was utilized to conduct qPCR with 2^−△△CT^ calculation. GAPDH served as control.

### Cell transfection

Specific shRNAs targeting PRR34-AS1 (sh/PRR34-AS1), as well as DDX3X (sh/DDX3X), Rab27a (sh/Rab27a) and negative control (sh/NC) were synthesized by Genechem (Shanghai, China). Besides, NC or PRR34-AS1 or Rab27a were obtained from RiboBio (Guangzhou, China). Cells were collected for further experiments after 48 h of transfection.

### 5-Ethynyl-2′-deoxyuridine (EdU)

Cell proliferation was assessed with the application of EdU kit (RiboBio, Guangzhou, China). Cells in 96-well plates were incubated to 90% confluence, and then cultured with 100 μL of 50 μM EdU diluent for 2 h. After fixation with 4% paraformaldehyde and 0.5% Triton X-100 treatment, cells were incubated in the dark with 100 μL of 1× Apollo® staining reaction. DAPI was applied for nuclear redyeing. Images were acquired by a laser confocal microscope.

### Colony formation

Cells were planted into 6-well plates (600 cells/well) for 2 weeks, followed by the treatment of 4% paraformaldehyde and 0.5% crystal violet. Finally, the number of colonies (> 50 cells) was counted.

### Wound healing

Cells were planted into 6-well plates until completely adhered to the wall, and then cells were scratched for cell culture. Images of wound healing were obtained at 0 h and 24 h after scratching, and the relative wound width was calculated.

### Transwell

Transwell chambers (8-μm pores, Corning, USA) pre-coated with Matrigel (BD, USA) were used for invasion assay. Cells were planted into the upper chamber, and the bottom chamber was filled with medium containing 10% FBS. After 24 h of incubation, cells were removed from the upper surface, and the invaded cells were subjected to the fixation with 4% paraformaldehyde for 10 min at room temperature, and stained in 0.5% crystal violet. The average number of invaded cells per field was assessed using a microscope.

### Western blot

Cells were cultured in RIPA buffer (Beyotime) for the extraction of proteins. BCA protein assay kit II (Beyotime) was performed for determining protein concentrations. The extracted proteins were subjected to the separation on 10% SDS-PAGE and then transferred onto PVDF membrane (Invitrogen), followed by being blocked with 3% BSA. Blots were incubated overnight at 4 °C with antibodies against E-cadherin (1/1000), N-cadherin (1/1000), Vimentin (1/1000), Slug (1/1000), Twist (1/1000), GAPDH (1/1000), CD9 (1/2000), CD63 (1/2000), HSP70 (1/1000), TSG101 (1/1000), DDX3X (1/1000), VEGF (1/1000), TGF-β (1/1000) and Rab27a (1/1000) and all these antibodies were procured from Abcam. Next, blots were incubated with horseradish peroxidase (HRP)-labeled secondary antibodies. The blot signals were measured through enhanced chemiluminescence reagents.

### Immunofluorescence staining

Cells were treated with 4% paraformaldehyde and 0.5% Triton X-100 for fixation and permeabilization, and then blocked in 3% bovine serum albumin. Subsequently, cells were cultured overnight at 4 °C with primary antibodies and cultured for 1 h at 37 °C with specific secondary antibodies. After washing, DAPI was utilized for nuclear redyeing. The images of Immunofluorescence staining were obtained under a confocal microscope.

### Exosome isolation

Exosomes were separated from cells using Ribo exosome isolation reagent (RiboBio, Guangzhou, China) based on the user manual. Cells were centrifuged at 2000×*g* for 30 min, and mixed with Ribo exosome solution reagent. The mixtures were refrigerated at 4 °C overnight, followed by centrifugation at 1500×*g* for 30 min. Exosomes were obtained after removing the supernatants.

### Exosome labeling

PKH67 (1 μM, Sigma-Aldrich) was commercially obtained to label the exosomes according to the supplier’s protocol. Cell nuclear was double-stained by DAPI. Images were observed under a laser scanning microscope.

### Nanoparticle tracking analysis (NTA)

The concentration and distribution of exosome size was measured using ZetaView PMX 10 (Particle Metrix, Germany). Exosomes were re-suspended in granular PBS and added to the instrument. Each sample was recorded in 5 videos to confirm the numbers and size distribution of exosomes.

### Transmission electron microscopy (TEM)

Cells were centrifuged and treated with 2.5% glutaraldehyde and then treated by osmium tetroxide. Next, cells were embedded with epoxy resin and cut into sections at a thickness of 100 nm, followed by the treatment of uranyl acetate and lead citrate. The images of microscopic samples were captured using a transmission electron microscope.

### Fluorescence in situ hybridization (FISH)

Cy3-labeled PRR34-AS1 probe was synthesized (RiboBio, Guangzhou, China). A FISH Kit (RiboBio) was applied for assessing the subcellular localization of PRR34-AS1 base on the manufacturer’s instructions.

### Subcellular fractionation

Subcellular isolation of RNAs was performed with the application of Cytoplasmic and Nuclear RNA Purification Kit which was procured from Norgen (Thorold, ON, Canada). Nuclear and cytoplasmic fractions were measured via RT-qPCR.

### Luciferase reporter assay

For Rab27a promoter analyses, the sequence of Rab27a promoter was firstly sub-cloned into pGL3-basic reporter vectors (Promega, Madison, WI, USA). After the co-transfection of downregulated PRR34-AS1 and plasmids, the activity of Rab27a promoter was evaluated using a luciferase assay kit (Promega).

### RNA-binding protein immunoprecipitation (RIP)

Magna RNA-binding protein immunoprecipitation kit (Millipore, USA) was utilized to assess the binding between RNAs. Cell lysates were cultured with RIP buffer and antibodies (anti-Ago2, anti-SNRNP70 and anti-DDX3X) conjugated with magnetic beads for 1 h. The enrichment of RNAs was evaluated by RT-qPCR.

### RNA pull down assay

Pierce Magnetic RNA–Protein Pull-down kit (Thermo fisher scientific) was used in this assay in the light of supplier’s instructions. PRR34-AS1 sense and PRR34-AS1 antisense were in vitro transcripted and labeled. Biotin-labeled PRR34-AS1 (50 pmol) was cultured with streptavidin magnetic beads (50 μL) with rotation, and then hatched in cell lysates. The eluted proteins were measured by western blot after mass spectrometry.

### Actinomycin D assay

After reaching 80% confluence, cells were treated with actinomycin D (4 µg/mL, Sigma-Aldrich, USA) to inhibit transcription. The relative mRNA levels were analyzed by RT-qPCR at the indicated time points.

### Statistical analysis

Data were processed by GraphPad Prism 6.0 Software and displayed as means ± standard deviation of three replicates. The Student’s t-test and two-way ANOVA were used to analyze differences between two groups or more than two groups. Data were defined as statistical significance if P < 0.05.

## Results

### PRR34-AS1 overexpression enhances the capacities of proliferation, migration, invasion and EMT of THLE-3 cells

**Fig. 1:**
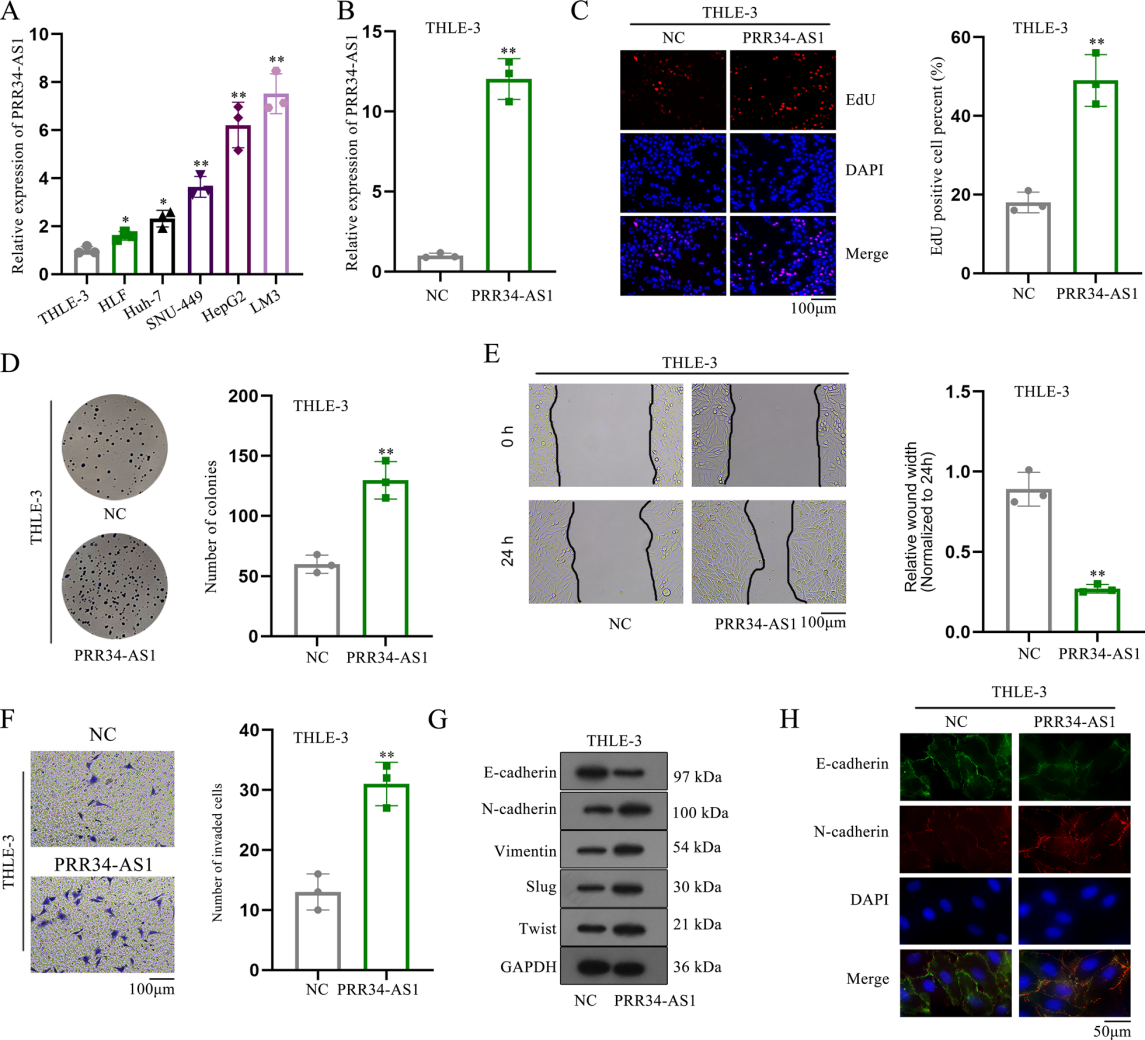
The overexpression of PRR34-AS1 facilitates the malignant phenotypes of THLE-3 cells. **A** PRR34-AS1 expression in HCC cells (HLF, Huh-7, SNU-449, HepG2 and LM3) was analyzed via RT-qPCR. Number of replicates (N) = 3. Data were analyzed via DGP PCR quantification. **B** The expression of PRR34-AS1 was analyzed by RT-qPCR in THLE-3 cells with PRR34-AS1 overexpression. N = 3. Data were analyzed via DGP PCR quantification. **C**, **D** EdU and colony formation assays were implemented to in THLE-3 cells after PRR34-AS1 overexpression. N = 3. Data were analyzed via ImageJ-win64. Scale bar = 100 µm. **E**, **F** THLE-3 cell migratory and invasive phenotypes was analyzed by wound healing and transwell assays after PRR34-AS1 overexpression. N = 3. Data were analyzed via ImageJ-win64. Scale bar = 100 µm. **G**, **H** The EMT process of THLE-3 cells was assessed by western blot analysis and immunofluorescence staining after PRR34-AS1 overexpression. N = 3. Scale bar = 50 µm. *P < 0.05, **P < 0.01

First, GEPIA 2 (http://gepia2.cancer-pku.cn) database was applied to assess PRR34-AS1 expression in liver hepatocellular carcinoma (LIHC) tissues. PRR34-AS1 exhibited a markedly high expression level in LIHC tissues (Additional file 1: Fig. S1A). Consistently, RT-qPCR analysis indicated that PRR34-AS1 was highly expressed in HCC cells including HLF, Huh-7, SNU-449, HepG2 and LM3 in comparison to THLE-3 cells (Fig. 1A). Since HepG2 and LM3 cells presented the highest PRR34-AS1 expression among the selected cells, both HepG2 and LM3 cells were selected for subsequent assays. We stably silenced PRR34-AS1 expression via transfecting two shRNAs targeting PRR34-AS1 into HepG2 and LM3 cells (Additional file 1: Fig. S1B). As shown in EdU and colony formation assays, PRR34-AS1 depletion reduced the percent of EdU positive cells and the number of colonies (Additional file 1: Fig. S1C, D). Meanwhile, the relative wound width was obviously increased when PRR34-AS1 was knocked down, suggesting that PRR34-AS1 silencing inhibited cell migratory ability in HCC (Additional file 1: Fig. S1E). Additionally, the number of invaded cells was also decreased in PRR34-AS1-silenced HepG2 and LM3 cells (Additional file 1: Fig. S1F). Moreover, we also used western blot to evaluate the role of PRR34-AS1 depletion on EMT in HCC cells. It turned out that PRR34-AS1 absence caused an upregulation in the level of epithelium marker (E-cadherin) whereas diminished the levels of mesenchymal markers (N-cadherin and Vimentin) and EMT transcription factors (Slug and Twist), implying that PRR34-AS1 silencing suppressed the EMT process in HCC cells (Additional file 1: Fig. S1G). Collectively, PRR34-AS1 enhances cell proliferative, migratory, invasive and EMT phenotypes in HCC.

We next up-regulated PRR34-AS1 expression in THLE-3 cells (Fig. 1B) and found that the EdU positive stained cells were sharply increased in THLE-3 cells after PRR34-AS1 overexpression (Fig. 1C). Likewise, PRR34-AS1 overexpression led to an augment on the number of colonies (Fig. 1D), suggesting that PRR34-AS1 up-regulation facilitated the proliferation ability of THLE-3 cells. Besides, we found that the relative wound width was shorter in PRR34-AS1 groups compared to NC group, implying that up-regulated PRR34-AS1 promoted the migratory capacity of THLE-3 cells (Fig. 1E). Simultaneously, transwell assays disclosed that PRR34-AS1 overexpression enhanced the invasive capacity of THLE-3 cells (Fig. 1F). Additionally, we also assessed the influence of PRR34-AS1 up-regulation on EMT in THLE-3 cells. E-cadherin level was obviously reduced whereas the levels of N-cadherin, Vimentin, Slug and Twist were enhanced in PRR34-AS1-up-regulated THLE-3 cells (Fig. 1G). Similarly, the intensity of E-cadherin was weakened whereas N-cadherin intensity was strengthened in THLE-3 cells when PRR34-AS1 was overexpressed, implying that PRR34-AS1 overexpression accelerated EMT in THLE-3 cells (Fig. 1H). Taken together, PRR34-AS1 up-regulation facilitated the malignant capacities of THLE-3 cells.

### Enhanced cell proliferative, migratory, invasion and EMT abilities are observed in co-cultured HCC and THLE-3 cells

**Fig. 2:**
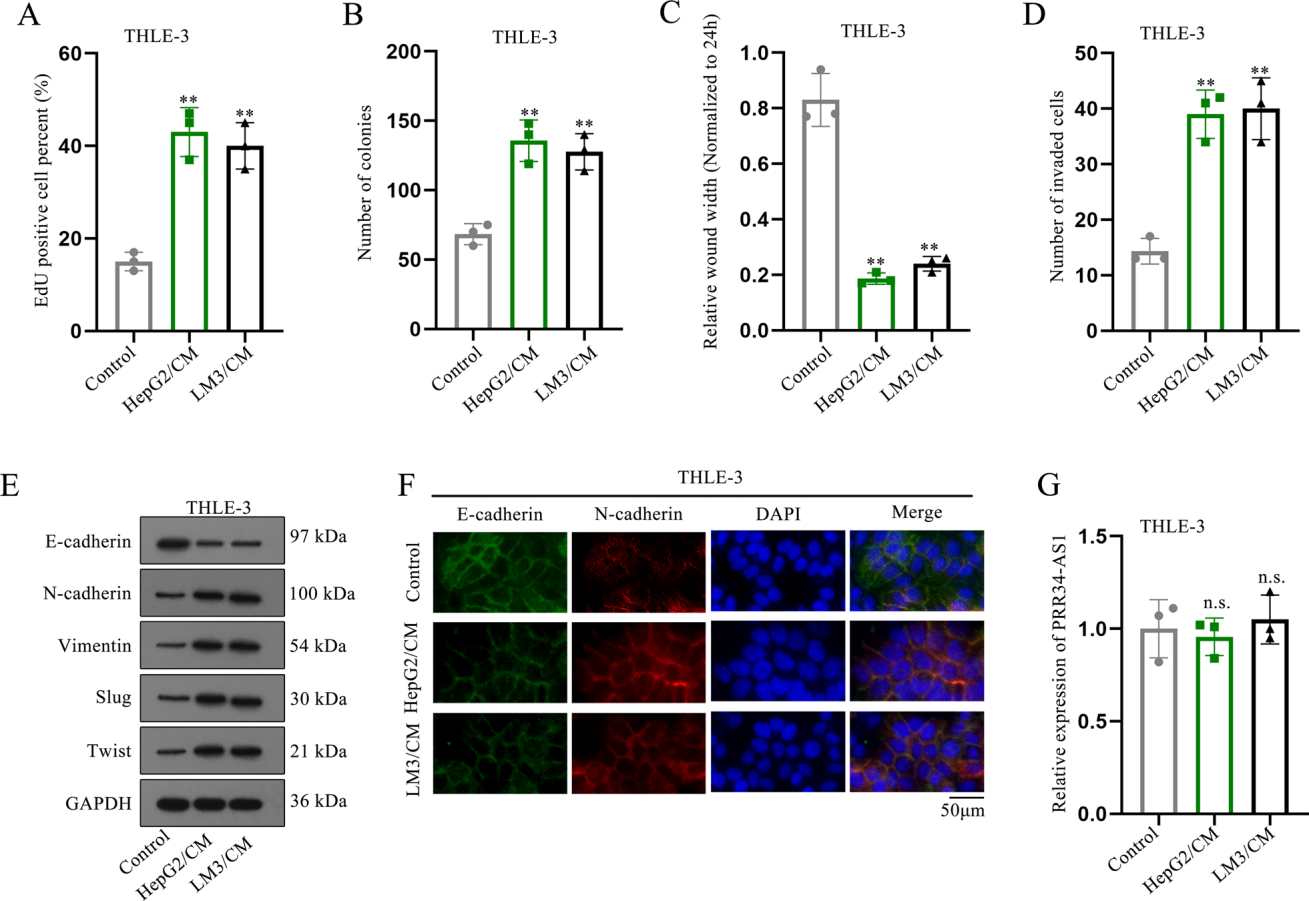
Increased cell proliferative, migratory, invasive and EMT capacities are observed in THLE-3 cells co-cultured with HCC cells. **A**, **B** EdU and colony formation assays were implemented to assess the proliferation of THLE-3 cells co-cultured with HCC cells. N = 3. **C**, **D** Wound healing and transwell assays were performed to assess the migration and invasion of THLE-3 cells co-cultured with HCC cells. N = 3. **E**, **F** The EMT process of THLE-3 cells co-cultured with HCC cells was measured by western blot analysis and immunofluorescence staining. N = 3. Scale bar = 50 µm. **G** PRR34-AS1 expression in THLE-3 cells co-cultured with HCC cells was analyzed via RT-qPCR. N = 3. Data were analyzed via DGP PCR quantification. **P < 0.01, n.s. meant no significance

Then, we wondered whether HCC cells could secrete exosomal PRR34-AS1 and transmit them to THLE-3 cells, leading to the promotion of cell proliferation, migration, invasion and EMT in THLE-3 cells. After the co-culture of HepG2 or LM3 cells with THLE-3 cells, we performed functional assays in co-cultured cells. Cell proliferation ability was enhanced in co-cultured cells relative to THLE-3 cells (Fig. 2A, B). Consistently, the potential of cell migration and invasion was markedly promoted in co-cultured cells (Fig. 2C, D). Moreover, we found that E-cadherin level was reduced while the level of N-cadherin, Vimentin, Slug and Twist were elevated in co-cultured cells (Fig. 2E). Meanwhile, the data from immunofluorescence staining also uncovered that the co-cultured cells had a stronger EMT capacity than THLE-3 cells (Fig. 2F). However, PRR34-AS1 expression had no marked change in co-cultured cells (Fig. 2G), which excluded the hypothesis of exosomal PRR34-AS1 in HCC cells. Collectively, the increased cell malignant phenotypes were observed in co-cultured HCC and THLE-3 cells.

### HCC cell-secreted exosomes promote cell malignant phenotypes

**Fig. 3:**
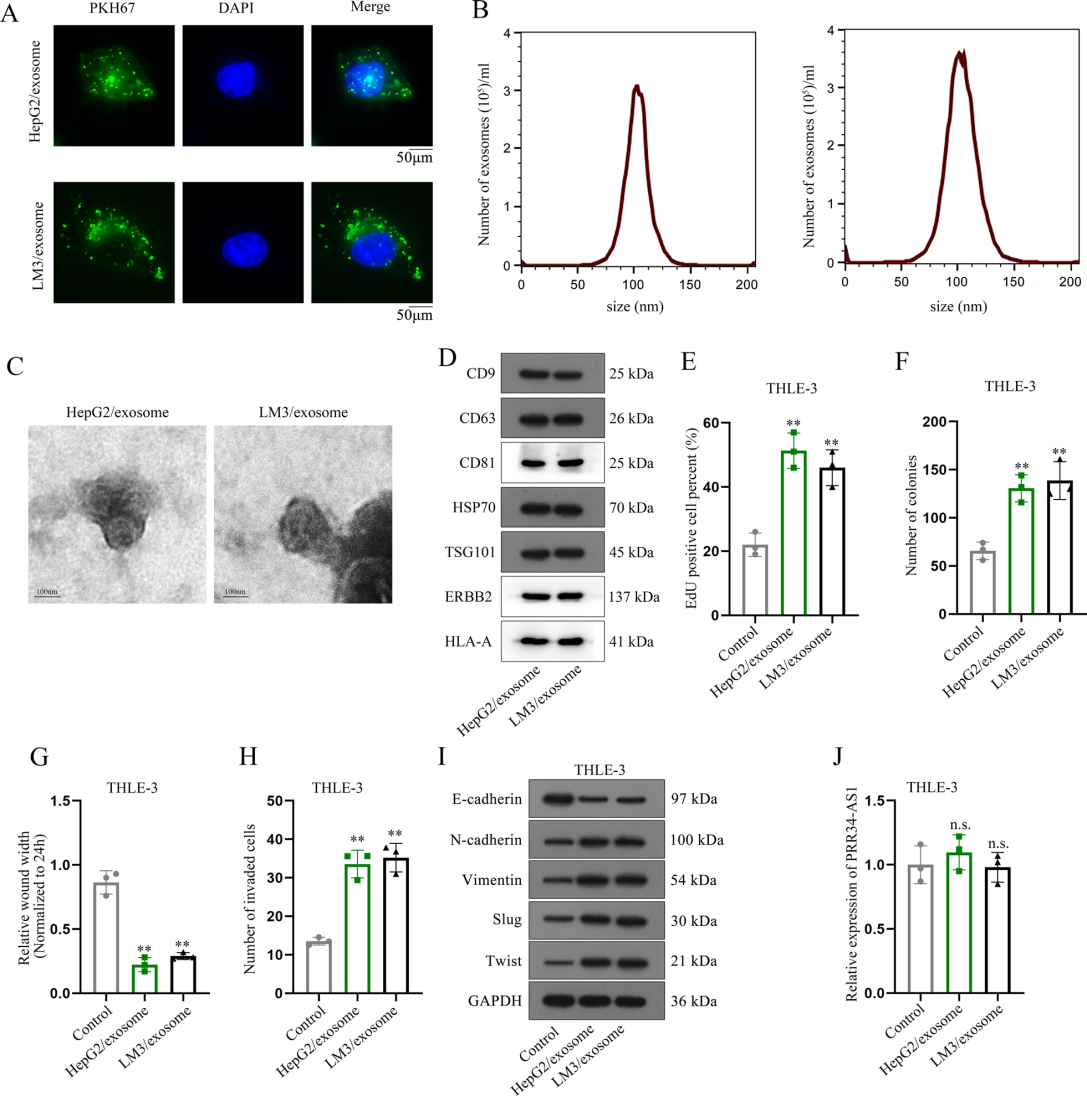
HCC cell-secreted exosomes facilitate cell malignant phenotypes. **A** PKH67 staining labeled HCC cell-secreted exosomes (HepG2/exosome and LM3/exosome). N = 3. Scale bar = 50 µm. **B** NTA measured the number and distribution size of exosomes. N = 3. **C** The images of HCC cell-secreted exosomes were observed under TEM. N = 3. Scale bar = 100 nm. **D** The levels of exosome-related proteins (CD9, CD63, HSP70 and TSG101) and extracellular vesicle-related proteins (CD81, ERBB2 and HLA-A) in HCC cell-secreted exosomes was measured by western blot. N = 3. **E**, **F** proliferation assays were conducted to assess the proliferative ability of THLE-3 cells treated with HCC cell-secreted exosomes. N = 3. **G**, **H** Wound healing and transwell assays were implemented to assess the migratory and invasive abilities of THLE-3 cells treated with HCC cell-secreted exosomes. N = 3. **I**, **J** Western blot and immunofluorescence staining were implemented to analyze the EMT process of THLE-3 cells treated with HCC cell-secreted exosomes. N = 3. Data were analyzed via DGP PCR quantification. **P < 0.01, n.s. meant no significance

Then, we speculated the existences of other exosome transmitted from HCC cells to THLE-3 cells. Exosomes from HCC cells were isolated and labeled with PKH67 dye (green). The result showed that PKH67-labeled exosomes dispersed in HCC cell cytoplasm (Fig. 3A). Besides, we found that exosomes ranged from 50 to 150 nm in diameter (Fig. 3B). Moreover, we found that the exosomes under TEM had the typically round or cup-shaped morphology (Fig. 3C). Additionally, western blot analysis further confirmed that HCC cells had abundant exosomal proteins (CD9, CD63, HSP70 and TSG101) and extracellular vesicle proteins (CD81, ERBB2 and HLA-A) (Fig. 3D). All above results validated the existence of exosomes in HCC cells. Subsequently, we treated HCC cell-secreted exosomes into THLE-3 cells to observe the changes of their cellular phenotypes. As shown in Fig. 3E, F, the proliferation ability was obviously increased in THLE-3 cells (Fig. 3E, F). Meanwhile, the capacities of migration and invasion were apparently enhanced in THLE-3 cells treated with HCC cell-secreted exosomes (Fig. 3G, H). Moreover, we found that the EMT process was also accelerated in THLE-3 cells treated with HCC cell-secreted exosomes (Fig. 3I). Expectedly, the expression of PRR34-AS1 had no significance change in THLE-3 cells treated with HCC cell-secreted exosomes (Fig. 3J). Collectively, HCC cell-secreted exosomes enhanced cell proliferation, migration, invasion and EMT.

### PRR34-AS1 regulates exosomal protein Rab27a in HCC cells

**Fig. 4:**
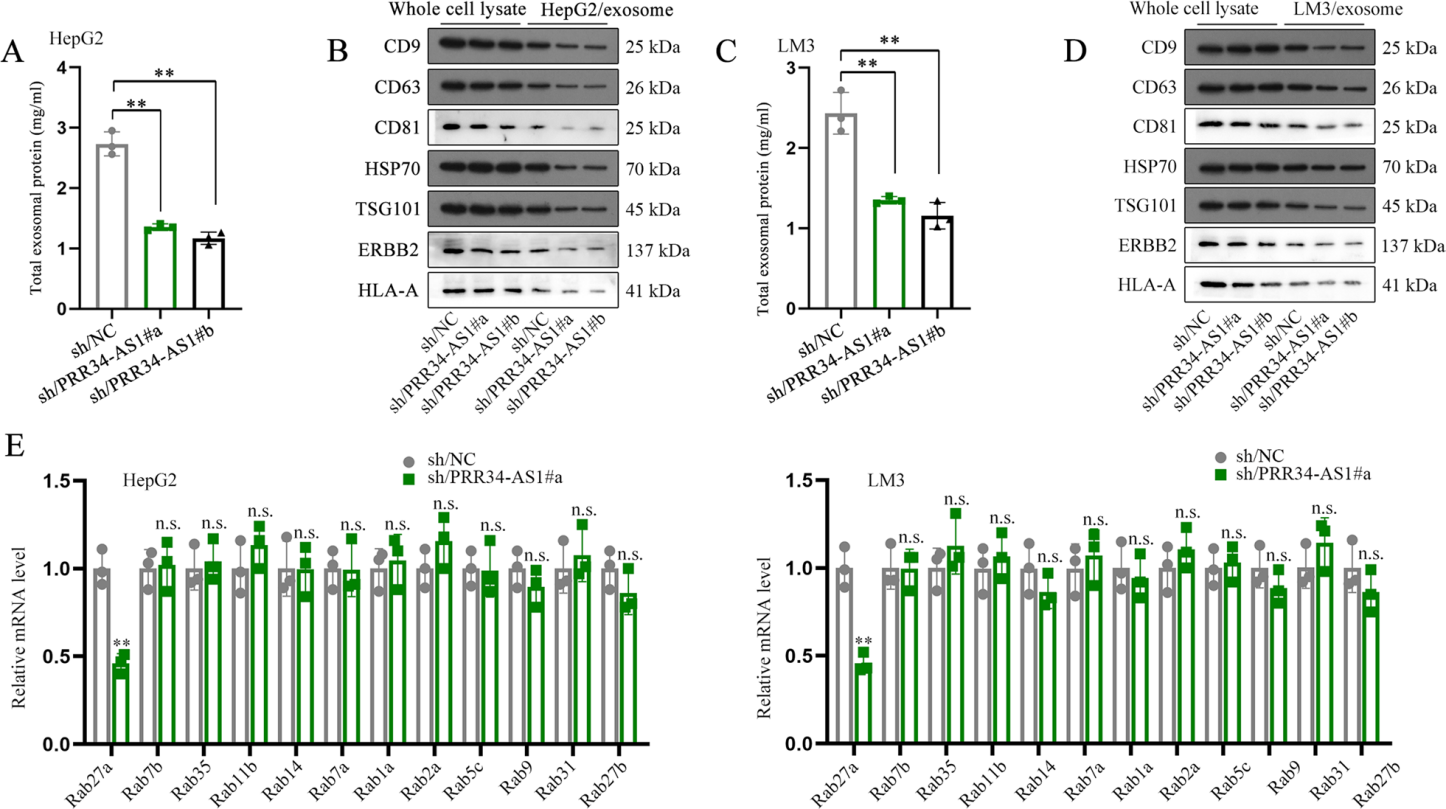
PRR34-AS1 regulates exosomal protein Rab27a in HCC cells. **A** Total exosomal protein in HepG2 cells was analyzed using RT-qPCR after PRR34-AS1 silencing. N = 3. **B** The level of exosome markers and extracellular vesicle-related proteins in HepG2 cell-secreted exosomes was analyzed using western blot after PRR34-AS1 silencing. N = 3. **C** Total exosomal protein in LM3 cells was analyzed using RT-qPCR after PRR34-AS1 silencing. N = 3. **D** The level of exosome markers and extracellular vesicle-related proteins in LM3 cell-secreted exosomes was analyzed using western blot after PRR34-AS1 silencing. N = 3. **E** Relative mRNA levels of exosomal proteins in HCC cells were analyzed using RT-qPCR after PRR34-AS1 silencing. N = 3. Data were analyzed via DGP PCR quantification. **P < 0.01, n.s. meant no significance

We extracted the exosomes secreted from HCC cells and found that the level of total exosomal protein, exosome markers (CD9, CD63, HSP70 and TSG101) and extracellular vesicle proteins (CD81, ERBB2 and HLA-A) was obviously decreased when PRR34-AS1 was downregulated (Fig. 4A–D). Therefore, we speculated that the level of HCC cell-secreted exosomal proteins might be regulated by PRR34-AS1. We then analyzed the level of exosomal proteins in HCC cells treated with downregulated PRR34-AS1. The level of Rab27a was substantially inhibited in HCC cells after PRR34-AS1 silencing and other exosomal proteins had no marked changes (Fig. 4E). Taken together, PRR34-AS1 regulated exosomal protein Rab27a in HCC cells.

### PRR34-AS1 interacts with DDX3X to regulate Rab27a mRNA stability

**Fig. 5:**
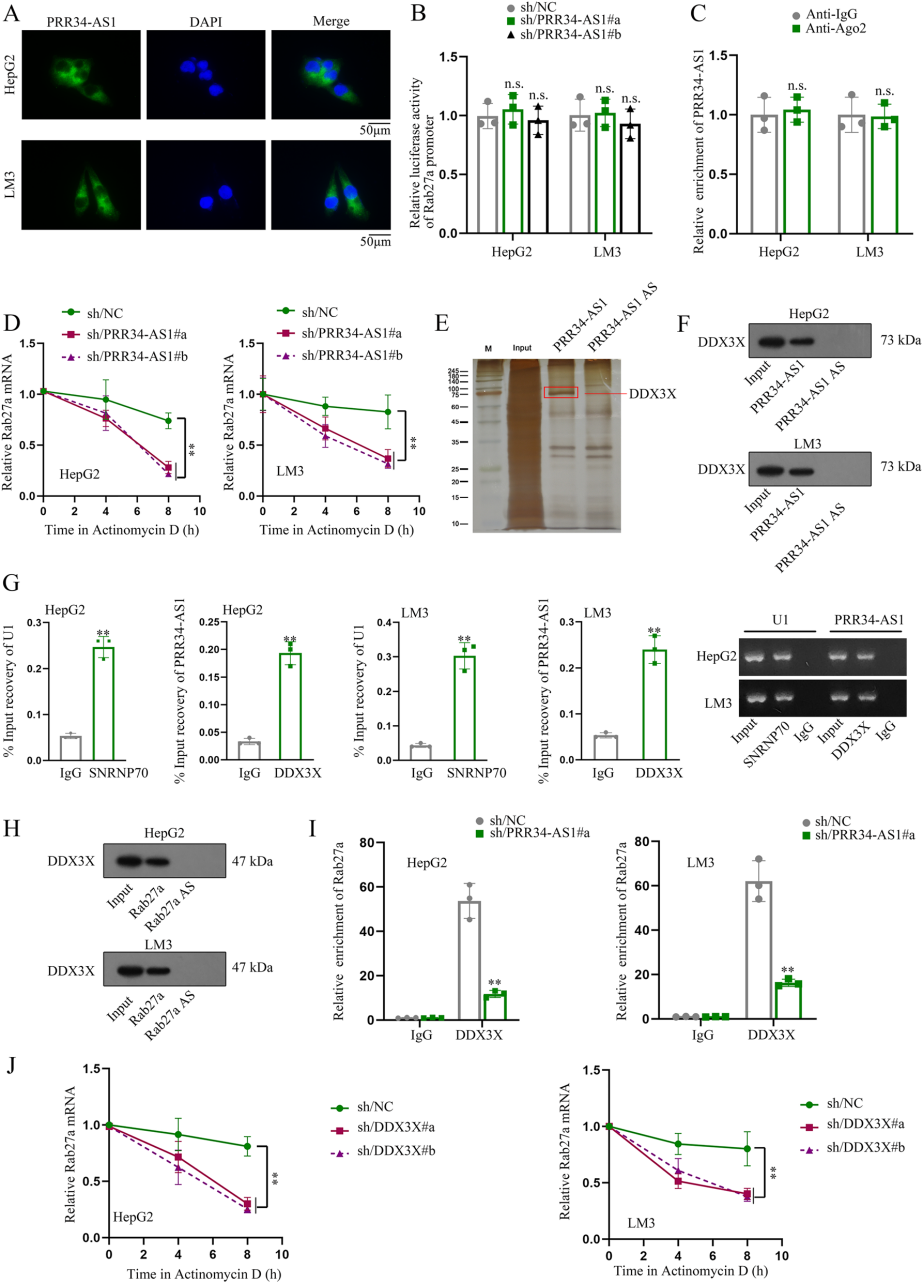
PRR34-AS1 interacts with DDX3X to regulate the stability of Rab27a mRNA. **A** The localization of PRR34-AS1 was assessed through FISH assay. N = 3. Scale bar = 50 µm. **B** Luciferase reporter assay was implemented to detect the activity of Rab27a promoter with PRR34-AS1 silencing. N = 3. **C** The combination between PRR34-AS1 and Ago2 in HCC cells was assessed by RIP assay. N = 3. Data were analyzed via DGP PCR quantification. **D** Actinomycin D treatment was implemented to evaluate the stability of Rab27a mRNA in HepG2 and LM3 cells via RT-qPCR after PRR34-AS1 silencing. N = 3. **E**, **F** RNA pull down assay was conducted to analyze the protein partner of PRR34-AS1, and the combination of PRR34-AS1 and DDX3X via western blot. N = 3. **G** The combination of PRR34-AS1 and DDX3X was evaluated via RIP assay. N = 3. Data were analyzed via DGP PCR quantification. **H** RNA pull down assay detected the combination of Rab27a and DDX3X. N = 3. **I** The combination of Rab27a and DDX3X in HCC cells was assessed by RIP assays after PRR34-AS1 silencing. N = 3. Data were analyzed via DGP PCR quantification. **J** The stability of Rab27a mRNA in HepG2 and LM3 cells was analyzed via RT-qPCR after DDX3X silencing. N = 3. **P < 0.01, n.s. meant no significance

To uncover the regulatory mechanism of PRR34-AS1 in HCC cells, we firstly assessed the cellular distribution of PRR34-AS1 in HCC cells. As shown in Fig. 5A and Additional file 2: Fig. S2A, PRR34-AS1 was primarily distributed in cell cytoplasm, indicating that PRR34-AS1 might post-transcriptionally regulate Rab27a in HCC cells. Meanwhile, luciferase reporter assays further confirmed this point, as indicated that PRR34-AS1 could not affect the luciferase activity of Rab27a promoter (Fig. 5B). It is well-acknowledged that lncRNA acts as a ceRNA to regulate cancer progression at post-transcriptional levels. We wondered whether PRR34-AS1 existed in the RNA-induced silencing complex (RISC) to bind to miRNAs and thereby regulated Rab27a expression. However, the outcome from RIP assays dispelled this hypothesis, as indicated that the enrichment of PRR34-AS1 had no significant change in Anti-Ago2 groups (Fig. 5C). Thus we further treated protein synthesis inhibitor (actinomycin D) into HCC cells to assess Rab27a mRNA level. The half-life of Rab27a mRNA was obviously declined after PRR34-AS1 silencing, indicating the regulatory effect of PRR34-AS1 on the stability of Rab27a mRNA (Fig. 5D). Then, the electrophoretic gel indicated that one specific band appeared at approximately 73 kDa and we finally identified it as DDX3X protein (Fig. 5E). Furthermore, the combination between PRR34-AS1 and DDX3X was confirmed by western blot and RIP analyses (Fig. 5F, G). Intriguingly, we also found the combination between Rab27a and DDX3X in HCC cells via RNA pull down assays (Fig. 5H). Moreover, we found that the combination of Rab27a and DDX3X was lessened in HCC cells after the downregulation of PRR34-AS1 (Fig. 5I). Thus we conjectured that PRR34-AS1 might interact with DDX3X to regulate Rab27a mRNA stability. We knocked down the levels of DDX3X and revealed that the levels of Rab27a were markedly decreased in HCC cells after DDX3X depletion (Additional file 2: Fig. S2B, C). Meanwhile, the stability of Rab27a mRNA was also reduced when DDX3X was silenced in HCC cells treated with actinomycin D (Fig. 5J). Collectively, PRR34-AS1 interacted with DDX3X to mediate Rab27a mRNA stability.

### PRR34-AS1 facilitates THLE-3 cells proliferation, migration, invasion and EMT via elevating Rab27a expression to increase the exosome secretion of VEGF and TGF-β

**Fig. 6:**
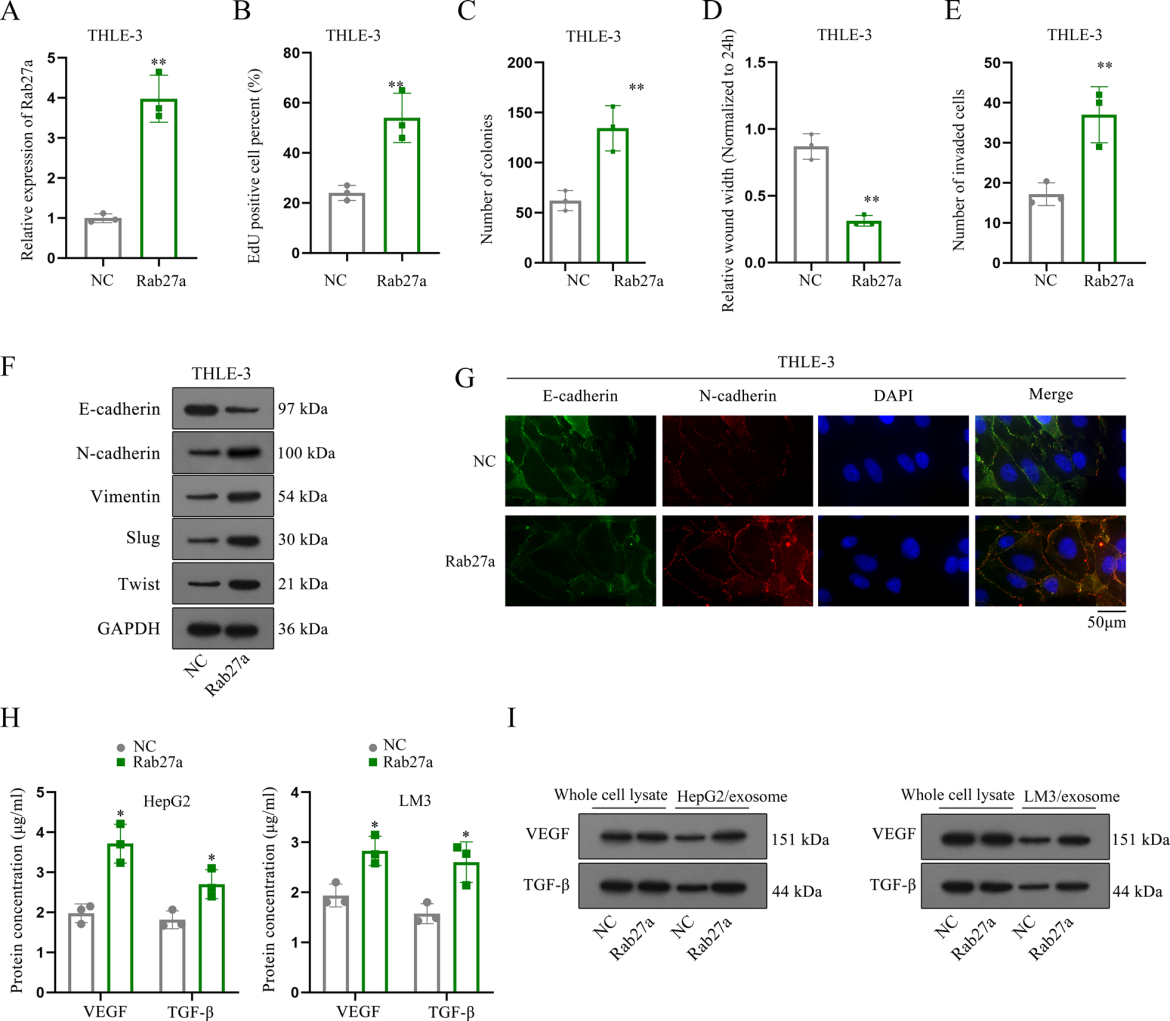
Rab27a facilitates THLE-3 cell malignant phenotypes via increasing the exosome secretion of VEGF and TGF-β. **A** Rab27a expression in THLE-3 cells was analyzed after Rab27a overexpression. N = 3. **B**, **C** The proliferation of THLE-3 cells was assessed via EdU and colony formation assays after Rab27a overexpression. N = 3. **D**, **E** Cell migratory and invasive abilities were evaluated through wound healing and transwell assays after Rab27a overexpression. N = 3. **F**, **G** The EMT process of THLE-3 cells was evaluated by western blot and immunofluorescence staining after Rab27a overexpression. N = 3. Scale bar = 50 µm. **H**, **I** The protein concentration of VEGF and TGF-β in THLE-3 cells was analyzed via RT-qPCR and western blot analyses with Rab27a overexpression. N = 3. *P < 0.05, **P < 0.01

Next, we investigated the role of Rab27a in THLE-3 cells. We overexpressed Rab27a expression in THLE-3 cells and observed that the proliferation ability was obviously enhanced when Rab27a was up-regulated in THLE-3 cells (Fig. 6A–C). In parallel, the capacities of migration and invasion were promoted in THLE-3 cells after Rab27a up-regulation (Fig. 6D, E). Moreover, we found that Rab27a overexpression elevated the EMT process in THLE-3 cells (Fig. 6F, G). It has been reported that Rab27a promotes tumor growth via increasing VEGF and TGF-β secretion in vitro [22]. Herein, we also found that the protein concentration of VEGF and TGF-β in THLE-3 cells as well as their protein levels in HCC cell-secreted exosomes was increased after Rab27a up-regulation (Fig. 6H, I). All above results suggested that Rab27a increased VEGF and TGF-β secretion in HCC cells and transmitted them into THLE-3 cells to elevate cell proliferative, migratory and invasive phenotypes as well as EMT.

To explore the effect of PRR34-AS1 and Rab27a on the malignant phenotypes of THLE-3 cells, rescue experiments were arranged after the verification of the knockdown efficiency of Rab27a (Additional file 3: Fig. S3A). As indicated in Additional file 4: Fig. S4A, B, Rab27a silencing reversed the enhanced proliferation ability in THLE-3 cells after PRR34-AS1 overexpression. Besides, it was found that the enhanced migration and invasion capacities induced by PRR34-AS1 up-regulation were abolished by co-transfection of sh/Rab27a#a (Additional file 4: Fig. S4C, D). Moreover, the decreased protein level of E-cadherin and the enhanced levels of N-cadherin, Vimentin, Slug and Twist in THLE-3 cells transfected with PRR34-AS1 overexpression was overturned by co-transfection of sh/Rab27a#a (Additional file 4: Fig. S4E). Meanwhile, the data of immunofluorescence assays further confirmed that Rab27a depletion abrogated the increased EMT process in THLE-3 cells after PRR34-AS1 augment (Additional file 4: Fig. S4F). Taken together, PRR34-AS1 facilitated THLE-3 cells proliferation, migration, invasion and EMT via elevating Rab27a expression.

## Discussion

In this research, we verified that lncRNA PRR34-AS1 recruited DDX3X to stabilize Rab27a mRNA and thereby promoted cell proliferative, migratory, invasive and EMT phenotypes in HCC. Meanwhile, PRR34-AS1 up-regulated Rab27a expression to increase the exosome secretion of VEGF and TGF-β in HCC cells and transmitted them into THLE-3 cells to accelerate the malignant phenotypes of THLE-3 cells. Our study revealed a novel function of PRR34-AS1 in regulating exosome secretion from HCC cells to THLE-3 cells, which provides a promising method for HCC treatment.

In recent years, lncRNAs have emerged as a pivot in the field of cancer research. LncRNA-D16366 is lowly expressed in HCC and might be an independent diagnostic and prognostic indicator for HCC [23]. RGMB-AS1 plays as a tumor suppressing role in HCC and is an independent favorable prognostic factor for patients with this disease [24]. All above studies demonstrated the anti-cancer effects of lncRNAs in HCC. In our research, we proved the oncogenic effect of lncRNA PRR34-AS1 in HCC. Furthermore, PRR34-AS1 exhibited a high expression level in HCC. PRR34-AS1 knockdown inhibited HCC cell proliferative, migratory, invasive and EMT abilities. Taken together, lncRNAs play an important role in the regulation of HCC progression.

Our study also found that PRR34-AS1 overexpression facilitated THLE-3 cell malignant phenotypes, suggesting the possibility of exosomes in HCC cells. Exosome-mediated communication in the tumor microenvironment contributes to HCC progression [5]. Exosome-transmitted lncRNA SENP3-EIF4A1 inhibits cell migration and invasion in HCC [25]. High expression of exosomal H19 enhances the proliferation and motility of HCC cells [26]. All these evidence suggested the regulation of exosomal lncRNA in HCC progression. In our study, we confirmed that the capacities of proliferation, migration, invasion and EMT were increased in THLE-3 cells co-cultured with HCC cells as well as in THLE-3 cells treated with HCC-secreted exosomes. However, PRR34-AS1 expression was not affected in THLE-3 cells co-cultured with HCC cells as well as in THLE-3 cells treated with HCC-secreted exosomes, which ruled out the existence of exosomal PRR34-AS1 in HCC.

As we all know, exosomes are a type of secretory membrane vesicle with the structural and biochemical characteristics of multivesicular endosomes [27]. Several Rab GTPases, including Rab27a, Rad27b, and Rab35, were previously found to play a crucial role in regulating exosome secretion and influencing cellular process [28]. It has been reported that lncRNA HOTAIR motivates exosome secretion via mediating RAB35 and SNAP23 in HCC [29]. In our study, we demonstrated that PRR34-AS1 silencing obviously reduced the expression of Rab27a. It has been reported that KIBRA modulates exosome secretion via repressing the proteasomal degradation of Rab27a [30]. All above results uncovered that PRR34-AS1 regulated Rab27a to affect exosome secretion in HCC cells.

Moreover, our study found that PRR34-AS1 interacted with DDX3X to regulate the stability of Rab27a mRNA. In line with our study, HHIP-AS1 inhibits HCC progression through recruiting HUR to stabilize HHIP mRNA [31]. FAM83A-AS1 expedites HCC progression by interacting with NOP58 to increase the mRNA stability of FAM83A [32]. After confirming the regulatory mechanism between PRR34-AS1 and Rab27a, we revealed that Rab27a accelerated cell malignant phenotypes in THLE-3 cells. More importantly, Rab27a up-regulation also promoted exosome secretion of VEGF and TGF-β, suggesting that PRR34-AS1 regulated Rab27a to promote the exosome secretion of VEGF and TGF-β and thereby transmitted them into THLE-3 cells to accelerate cell malignant phenotypes.

However, our study had some limitations. We didn’t unravel the detailed mechanism of Rab27a on regulating exosome secretion of VEGF and TGF-β, which will be addressed in the future.

## Supplementary Information

**Additional file 1: Figure S1.** PRR34-AS1 silencing inhibited HCC cell malignant phenotypes. **A** GEPIA 2 database analyzed PRR34-AS1 expression in LIHC tissues and normal tissues. **B** PRR34-AS1 expression in HCC cells was analyzed via RT-qPCR after PRR34-AS1 silencing. N = 3. Data were analyzed via DGP PCR quantification. **C**, **D** HCC cell proliferation was assessed by proliferation assays after PRR34-AS1 silencing. N = 3. **E**, **F** HCC cell migratory and invasive abilities was measured after PRR34-AS1 silencing. N = 3. **G** The EMT process of HCC cells was evaluated after PRR34-AS1 silencing. N = 3. *P < 0.05, **P < 0.01.

**Additional file 2: Figure S2.** DDX3X positively regulates Rab27a expression in HCC cells. **A** The cellular distribution of PRR34-AS1 in HCC cells was confirmed by subcellular fractionation assay. N = 3. **B**, **C** The mRNA and protein levels of DDX3X and Rab27a were analyzed in HCC cells transfected with sh/DDX3X#a/b. N = 3. **P < 0.01. Data were analyzed via DGP PCR quantification.

**Additional file 3: Figure S3.** Transfection efficiency of Rab27a in HCC cells. **A** Rab27a levels in HCC cells was detected by RT-qPCR and western bot analyses after Rab27a silencing. N = 3. **P < 0.01.

**Additional file 4: Figure S4.** PRR34-AS1 enhances THLE-3 cell malignant phenotypes via elevating the expression of Rab27a. **A**–**F** Rescue experiments were carried on in THLE-3 cells transfected with NC, PRR34-AS1, PRR34-AS1 + sh/Rab27a#a to assess cell proliferative, migratory and invasive as well as EMT phenotypes. N = 3. Scale bar = 50 µm. **P < 0.01.

## Data Availability

Research data are not shared.

## Abbreviations

HCC: Hepatocellular carcinoma
lncRNAs: Long non-coding RNAs
PRR34-AS1: PRR34 long non-coding RNA antisense RNA 1
DDX3X: DEAD-box helicase 3 X-linked
Rab27a: RAB27A, member RAS oncogene family
RT-qPCR: Quantitative real-time polymerase chain reaction
EdU: 5-Ethynyl-2′-deoxyuridine
RIP: RNA immunoprecipitation
GAPDH: Glyceraldehyde-3-phosphate dehydrogenase
SD: Standard deviation
FISH: Fluorescent in situ hybridization
TEM: Transmission electron microscopy
NTA: Nanoparticle tracking analysis
