# Supplementary figures and images for: PRR34-AS1 promotes exosome secretion of VEGF and TGF-β via recruiting DDX3X to stabilize Rab27a mRNA in hepatocellular carcinoma
Source: J Transl Med. 2022 Oct 27;20:491. doi: 10.1186/s12967-022-03628-9 (PMC9615160; doi:10.1186/s12967-022-03628-9)

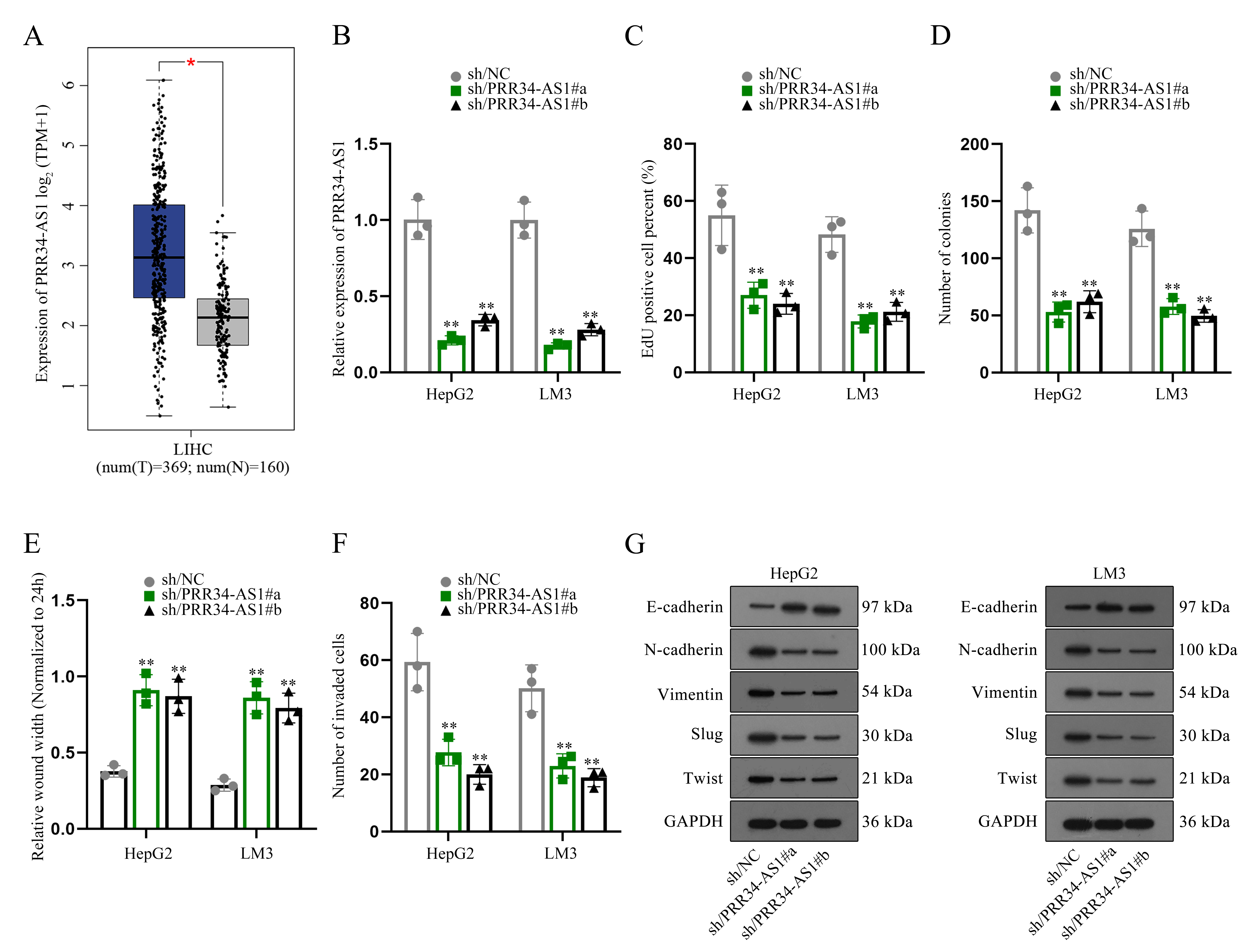

Supplement: Supplementary file 1 — Additional file 1: Figure S1. PRR34-AS1 silencing inhibited HCC cell malignant phenotypes. A GEPIA 2 database analyzed PRR34-AS1 expression in LIHC tissues and normal tissues. B PRR34-AS1 expression in HCC cells was analyzed via RT-qPCR after PRR34-AS1 silencing. N = 3. Data were analyzed via DGP PCR quantification. C, D HCC cell proliferation was assessed by proliferation assays after PRR34-AS1 silencing. N = 3. E, F HCC cell migratory and invasive abilities was measured after PRR34-AS1 silencing. N = 3. G The EMT process of HCC cells was evaluated after PRR34-AS1 silencing. N = 3. *P < 0.05, **P < 0.01. [file 12967_2022_3628_MOESM1_ESM.tif]

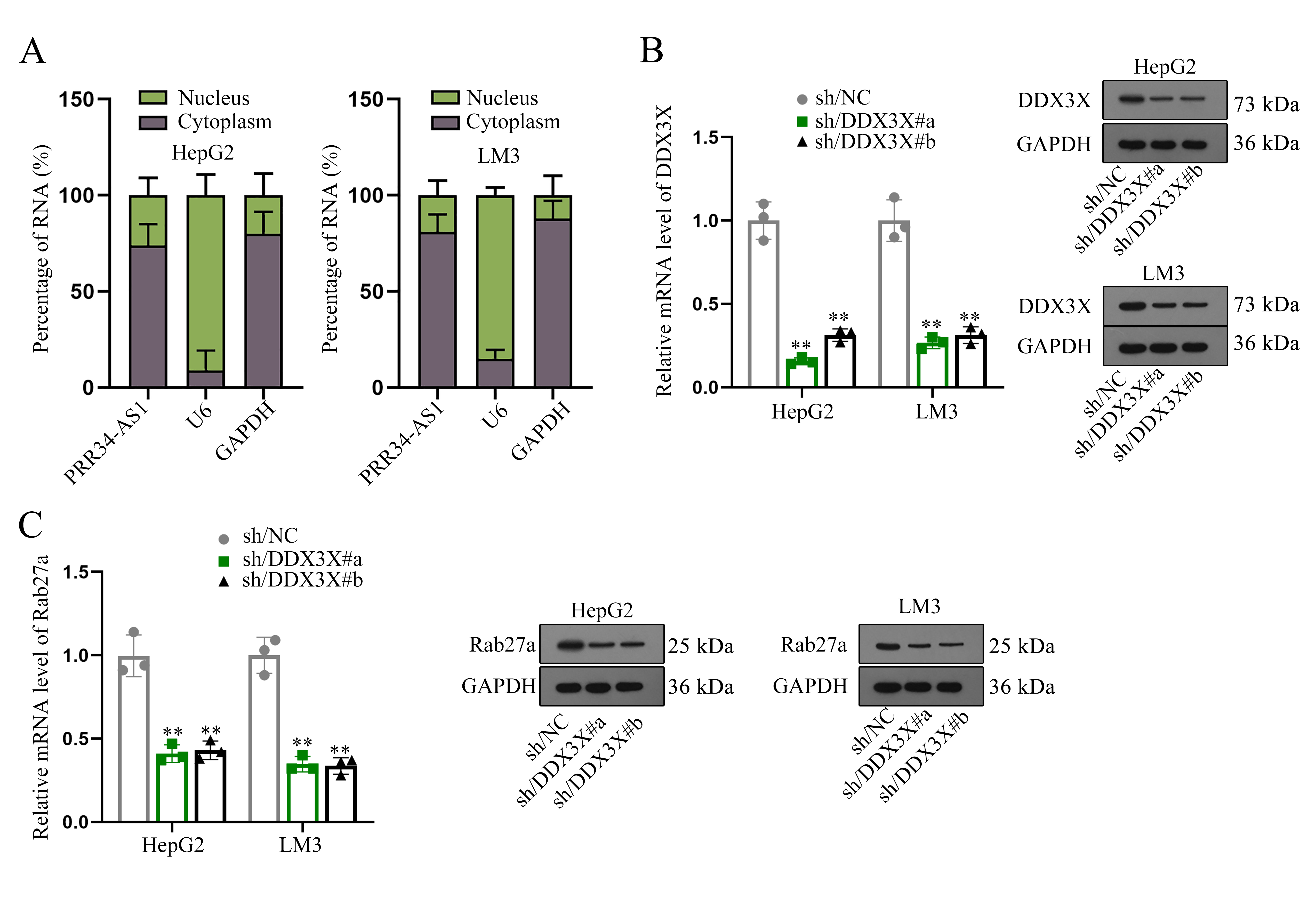

Supplement: Supplementary file 2 — Additional file 2: Figure S2. DDX3X positively regulates Rab27a expression in HCC cells. A The cellular distribution of PRR34-AS1 in HCC cells was confirmed by subcellular fractionation assay. N = 3. B, C The mRNA and protein levels of DDX3X and Rab27a were analyzed in HCC cells transfected with sh/DDX3X#a/b. N = 3. **P < 0.01. Data were analyzed via DGP PCR quantification. [file 12967_2022_3628_MOESM2_ESM.tif]

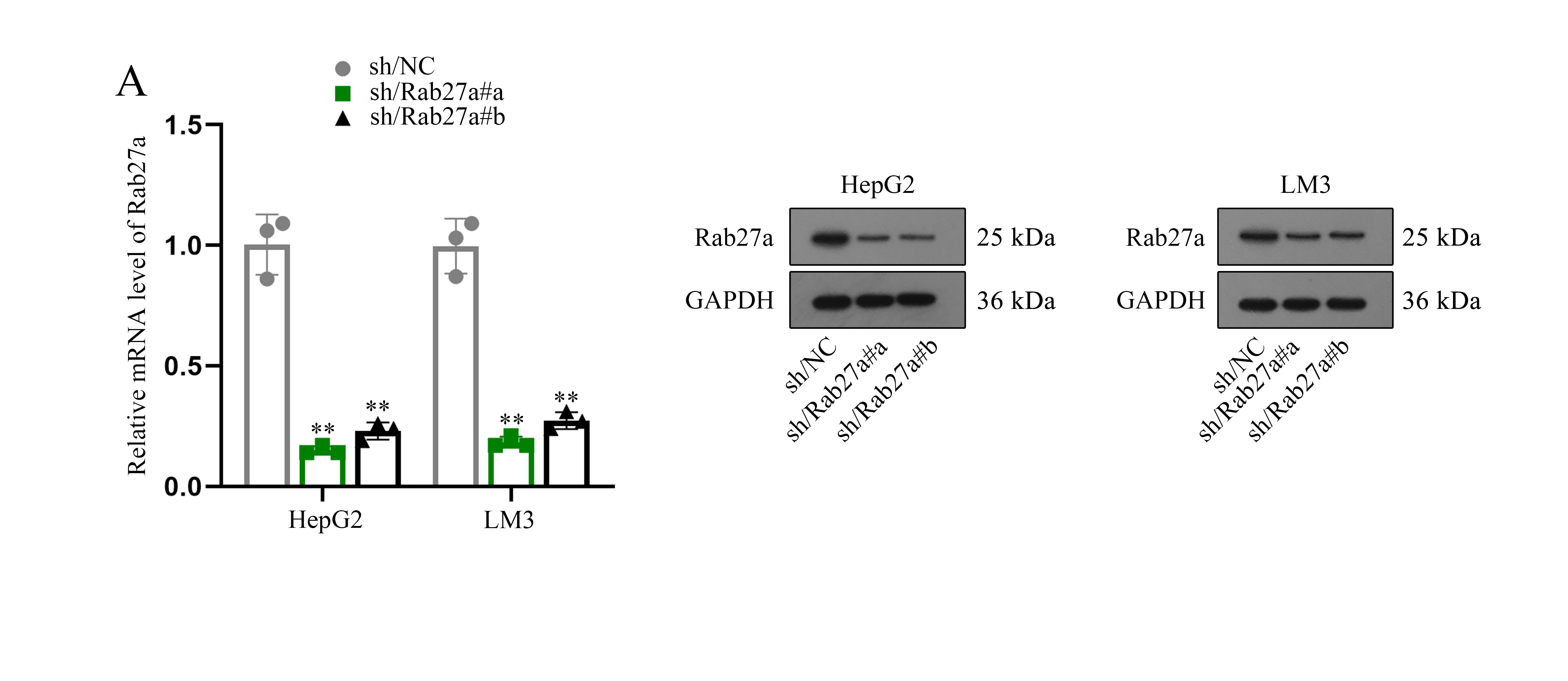

Supplement: Supplementary file 3 — Additional file 3: Figure S3. Transfection efficiency of Rab27a in HCC cells. A Rab27a levels in HCC cells was detected by RT-qPCR and western bot analyses after Rab27a silencing. N = 3. **P < 0.01. [file 12967_2022_3628_MOESM3_ESM.tif]

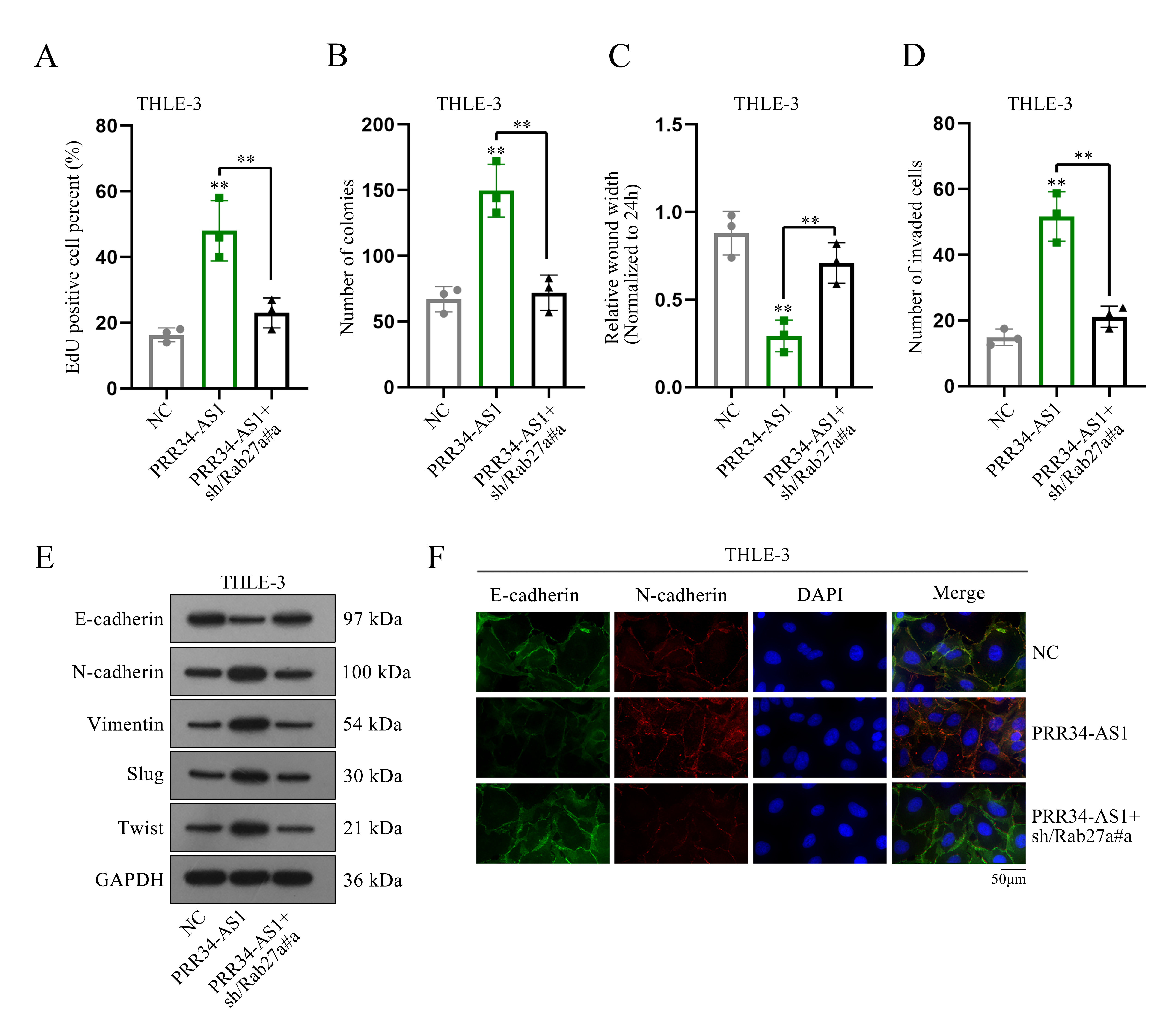

Supplement: Supplementary file 4 — Additional file 4: Figure S4. PRR34-AS1 enhances THLE-3 cell malignant phenotypes via elevating the expression of Rab27a. A–F Rescue experiments were carried on in THLE-3 cells transfected with NC, PRR34-AS1, PRR34-AS1 + sh/Rab27a#a to assess cell proliferative, migratory and invasive as well as EMT phenotypes. N = 3. Scale bar = 50 µm. **P < 0.01. [file 12967_2022_3628_MOESM4_ESM.tif]
